# CT-Derived Pectoralis Muscle Measurements and All-Cause Mortality in COPD

**DOI:** 10.3390/diagnostics16111645

**Published:** 2026-05-27

**Authors:** Ki Ryoung Lee, Kiook Baek, Jin Young Kim, Yong Sik Kwon, Euntaek Hwang, Byoungje Kim, Mu Sook Lee, Jung Hee Hong

**Affiliations:** 1 Department of Radiology, Keimyung University Dongsan Medical Center, 1035 Dalgubeol-daero, Dalseo-gu, Daegu 42601, Republic of Korea; 2 Department of Occupational and Environmental Medicine, Dongguk University Gyeongju Hospital, 87 Dongdae-ro, Gyeongju 38067, Republic of Korea; 3 Department of Preventive Medicine, Dongguk University, 87 Dongdae-ro, Gyeongju 38067, Republic of Korea; 4 Division of Cardiothoracic Imaging, Department of Radiology, Emory University, Atlanta, GA 30322, USA; 5 Division of Pulmonary Medicine, Department of Internal Medicine, Keimyung University Dongsan Medical Center, 1035 Dalgubeol-daero, Dalseo-gu, Daegu 42601, Republic of Korea

**Keywords:** chronic obstructive pulmonary disease, computed tomography, pectoralis muscle, sarcopenia, mortality

## Abstract

**Background/Objectives**: Skeletal muscle depletion is an important extrapulmonary manifestation of chronic obstructive pulmonary disease (COPD) and is associated with adverse clinical outcomes. Chest computed tomography (CT), which is frequently performed in patients with COPD, provides an opportunity for opportunistic assessment of thoracic muscle mass. However, the prognostic relevance of CT-derived pectoralis muscle measurements for long-term survival in COPD remains incompletely defined. This study aimed to evaluate the association between CT-derived pectoralis muscle measurements and all-cause mortality in patients with COPD and to compare the prognostic relevance of absolute muscle area and a height-adjusted index. **Methods**: In this retrospective cohort study, 245 patients with COPD who underwent chest CT were included. Pectoralis muscle area (PMA) was measured on a single axial image at the level of the fourth thoracic vertebra using a semi-automated segmentation method, and the pectoralis muscle index (PMI) was calculated by normalizing PMA to height squared. The primary endpoint was all-cause mortality. Multivariable Cox proportional hazards regression analyses were performed to assess the associations between muscle measurements and mortality, adjusting for age, sex, and selected clinical covariates. Hazard ratios (HRs) were expressed per 100-unit increase in PMA (mm^2^) and PMI. **Results**: During a mean follow-up of 5.31 ± 3.93 years, 178 deaths (72.7%) occurred. In multivariable analyses, higher PMA was significantly associated with a lower risk of all-cause mortality (HR per 100 mm^2^ increase, 0.951; 95% confidence interval [CI], 0.933–0.969; *p* < 0.001). Similarly, higher PMI was significantly associated with lower mortality (HR per 100-unit increase in PMI, 0.879; 95% CI, 0.834–0.925; *p* < 0.001). In sex-stratified analyses, these associations remained significant in men but not in women. **Conclusions**: CT-derived pectoralis muscle measurements were significantly associated with all-cause mortality in patients with COPD. Both absolute muscle area and height-adjusted indices demonstrated consistent prognostic value. Opportunistic assessment of thoracic muscle on routine chest CT may provide a useful imaging biomarker for risk stratification in COPD.

## 1. Introduction

Chronic obstructive pulmonary disease (COPD) is a leading cause of morbidity and mortality worldwide [1], with a substantial burden driven not only by persistent airflow limitation but also by its systemic nature and associated extrapulmonary manifestations [2,3]. Among these extrapulmonary manifestations, skeletal muscle dysfunction and sarcopenia are particularly important because they are associated with reduced exercise capacity, poorer quality of life, and worse prognosis in patients with COPD [4,5,6]. In this context, skeletal muscle depletion has been proposed as a key indicator of systemic disease burden and physiologic vulnerability [7,8].

CT-based assessment of skeletal muscle has attracted growing attention as an objective and reproducible approach to body composition evaluation [9,10,11]. Because chest CT is frequently performed in patients with COPD, it offers a practical opportunity to derive imaging biomarkers without additional examinations [12,13,14]. Among various skeletal muscle groups, the pectoralis muscle has been proposed as a useful imaging biomarker, as it is consistently included in routine chest CT scans and can be measured reproducibly at a standardized anatomical landmark, typically at the level of the fourth thoracic vertebra [15,16]. This enables opportunistic and quantitative assessment of muscle mass without additional imaging. Previous studies have demonstrated that CT-derived pectoralis muscle measurements are associated with clinically relevant features of COPD, and that reduced muscle mass is linked to increased mortality risk in patients with COPD [17,18,19]. These findings support the use of the pectoralis muscle as a surrogate marker of systemic muscle depletion and a potential prognostic imaging biomarker. Importantly, because skeletal muscle mass varies substantially among individuals depending on factors such as body size and sex, normalization approaches such as height-adjusted indices have been proposed to improve comparability across patients [20,21]. In line with this rationale, several studies have further characterized these associations in relation to disease severity, airflow limitation, and respiratory symptoms [22,23]. Furthermore, recent studies suggest that CT-derived body composition metrics may have prognostic value in COPD and related populations [24,25].

However, despite increasing interest in pectoralis muscle quantification, its role as a prognostic marker for long-term survival in COPD remains incompletely defined. In particular, it is unclear whether absolute muscle measures and height-adjusted indices provide comparable prognostic information, or whether one approach offers additional clinical value over the other [12,20].

Therefore, the aim of this study was to evaluate the association between CT-derived pectoralis muscle measurements and all-cause mortality in patients with COPD, and to compare the prognostic relevance of absolute pectoralis muscle area (PMA) and the height-adjusted pectoralis muscle index (PMI).

## 2. Materials and Methods

### 2.1. Study Population

**Figure 1:**
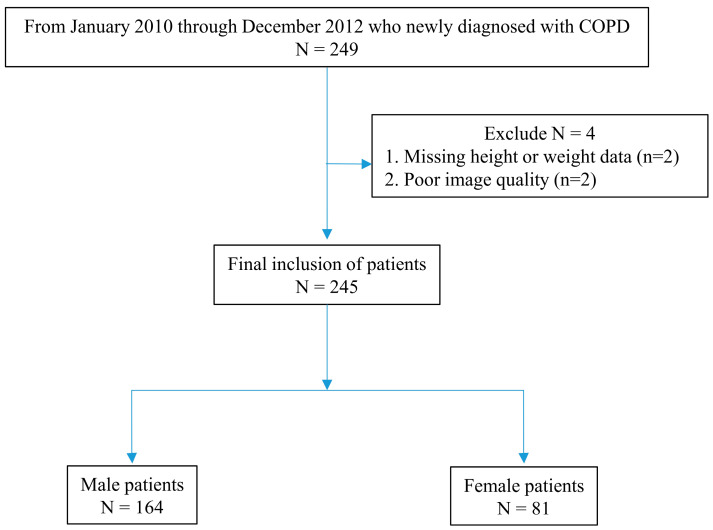
Enrollment of the study population.

From January 2010 to December 2012, we retrospectively collected 249 adult patients diagnosed with COPD who underwent chest CT at a single tertiary referral center. The diagnosis of COPD was made on the basis of the Global Initiative for Chronic Obstructive Lung Disease (GOLD) criteria [26]. Among patients with available chest CT examinations, the scan closest to the time of COPD diagnosis was selected as the baseline CT for analysis. The exclusion criteria were as follows: (1) missing height or weight data (*n* = 2), and (2) inadequate image quality that precluded accurate delineation of the pectoralis muscle (*n* = 2). After applying these criteria, a total of 245 patients (164 men and 81 women) were included in the final analysis (Figure 1).

### 2.2. CT-Based Pectoralis Muscle Quantification

Chest CT examinations were analyzed using a commercially available artificial intelligence platform (AVIEW Metrics; Coreline Soft, Seoul, Republic of Korea) to quantify pectoralis muscle morphology. When multiple chest CT scans were available during the follow-up period, the scan closest to the time of COPD diagnosis was selected for analysis.

For each patient, the T4 vertebral body was identified on coronal reformatted images, and a single axial image at the mid-vertebral level was selected for analysis. This anatomic landmark was chosen to ensure consistent cross-sectional visualization of the bilateral pectoralis muscles [12,13,14,15,16].

**Figure 2:**
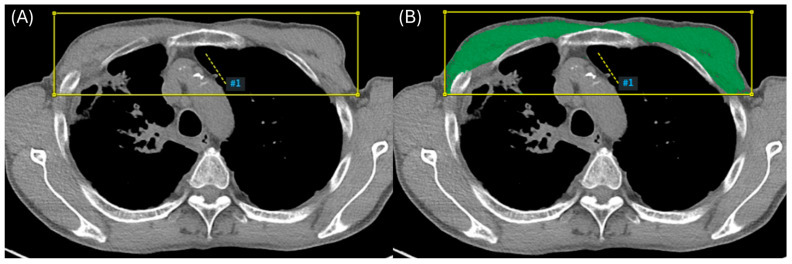
Representative axial CT images at the mid-T4 level from a 72-year-old man, demonstrating pectoralis muscle segmentation using a commercial software program (AVIEW Metrics, Coreline Soft, Seoul, Republic of Korea). (**A**) Original CT image. (**B**) Segmented image, with the green area highlighting the segmented pectoralis muscle. The yellow frame and dotted line indicate the selected axial plane at the mid-T4 level, and “#1” denotes the selected slice for analysis.

Pectoralis muscle area (PMA, mm^2^) was defined as the combined cross-sectional area of the right and left pectoralis muscles on a single axial image at the mid-T4 level, and segmentation was performed semi-automatically. All segmentations were visually reviewed by an experienced thoracic radiologist to ensure adequate image quality and to assess for obvious structural abnormalities of the pectoralis muscles, such as marked asymmetry, severe atrophy, post-surgical or traumatic changes, and metallic artifacts. No included patients demonstrated severe pectoralis muscle atrophy related to prior surgery or metallic materials that could interfere with measurements. Voxels within a predefined attenuation range of −30 to 160 Hounsfield units were initially selected to capture skeletal muscle tissue, after which the segmented regions were reviewed and manually refined by a chest radiologist (J.Y.K., 13 years of experience in chest CT interpretation). The final segmentation maps were independently verified by a second experienced thoracic radiologist (J.H.H., 11 years of clinical experience in chest CT interpretation) [12,13,14,15,16] (Figure 2). Both radiologists were blinded to all clinical and outcome data. Although interobserver agreement was not formally quantified, segmentation was performed using a standardized protocol with independent verification to enhance measurement reliability [27].

The pectoralis muscle index (PMI) was calculated by dividing PMA by the square of height in meters (PMI = PMA/height^2^ [m^2^]). This normalization was applied to account for inter-individual variability in muscle mass related to body size and to enhance comparability across patients, as commonly performed in body composition analysis.

### 2.3. Clinical Variables and Outcome Definition

Baseline clinical variables included age, sex, hypertension (HTN), diabetes mellitus (DM), asthma, coronary artery disease, cardiac disease, cancer, and inhaler use. Comorbidities were defined based on documented prior diagnoses in the electronic medical records and/or the use of corresponding medications at baseline. Inhaler use was defined as any use of inhaled respiratory medication (bronchodilator with or without corticosteroids) at baseline or during follow-up.

The primary endpoint was all-cause mortality. Mortality status was ascertained using administrative data obtained from the Ministry of the Interior and Safety, with records updated through March 2026. Additionally, hospital visit records were reviewed through March 2024 to determine the last follow-up date. Follow-up duration was defined as the interval from the date of baseline chest CT to the date of death or last follow-up. Death was treated as the event of interest, and patients who remained alive at the end of follow-up were censored. The hospital-based follow-up period was restricted to March 2024 in accordance with the approved IRB protocol, whereas mortality data were obtained from nationwide administrative records, allowing complete ascertainment of death events beyond the institutional follow-up period.

### 2.4. Ethical Statement

This retrospective study was approved by the institutional review board of Keimyung University Hospital (IRB number, DSMC 2024-04-009-013); the requirement for patients’ informed consent was waived due to the retrospective nature of the study and the use of anonymized data, which posed minimal risk to participants.

### 2.5. Statistical Analysis

Continuous variables are presented as mean ± standard deviation, and categorical variables as frequencies and percentages. Baseline characteristics were compared according to sex using the Welch Two Sample *t*-test for continuous variables and Pearson’s chi-squared test for categorical variables.

To evaluate the association between pectoralis muscle metrics and mortality, multivariable Cox proportional hazards regression analyses were performed using PMA and PMI as separate predictors in independent models. The analyses were adjusted for age, sex, hypertension, diabetes mellitus, asthma, coronary artery disease, cardiac disease, cancer, and inhaler use. Hazard ratios (HRs) and 95% confidence intervals (CIs) were reported. HRs for PMA and PMI were expressed per 100-unit increase in each variable to facilitate interpretability. Additional sex-stratified analyses were conducted to assess potential differences in the association between pectoralis muscle metrics and mortality according to sex.

As a sensitivity analysis, a random survival forest (RSF) was fitted with 1000 trees. PMA and PMI were entered as exposures of interest in separate models, with the same set of confounders as in the primary Cox analyses, and the analysis was repeated in the overall cohort and in each sex-specific subgroup. Model performance was quantified using the out-of-bag concordance index (C-index) and compared with that of the corresponding Cox models. The contribution of each variable to mortality prediction was quantified using permutation-based variable importance (VIMP), with higher values indicating greater predictive contribution; VIMP for the primary exposure was reported for each model, and the full VIMP for all covariates was presented.

All statistical analyses were performed using R version 4.3.2 (R Foundation for Statistical Computing, Vienna, Austria) with the survival package (version 3.7.0) for Cox proportional hazards modeling and the randomForestSRC package (version 3.6.2) for random survival forest analysis. A two-sided *p*-value of less than 0.05 was considered statistically significant.

## 3. Results

### 3.1. Baseline Characteristics

A total of 245 patients with COPD were included in the analysis, comprising 164 men (66.9%) and 81 women (33.1%). The mean age of the overall cohort was 70.2 ± 9.4 years. The mean follow-up duration was 5.31 ± 3.93 years, during which 178 deaths (72.7%) occurred. Patient enrollment is summarized in Figure 1.

Baseline characteristics stratified by sex are summarized in Table 1. Both PMA and PMI were significantly higher in men than in women (PMA: 3163.24 ± 938.57 mm^2^ vs. 2179.10 ± 564.59 mm^2^; PMI: 1164.04 ± 329.17 vs. 973.55 ± 249.25; both *p* < 0.001). Asthma was more frequent in women than in men (42.0% vs. 20.7%, *p* < 0.001). No statistically significant differences were observed between men and women in age, follow-up duration, death, HTN, DM, coronary artery disease, cardiac disease, cancer, or inhaler use.

### 3.2. Association Between PMA, PMI and Mortality

In the multivariable Cox proportional hazards model including PMA, higher PMA was significantly associated with a lower risk of all-cause mortality (HR per 100 mm^2^ increase, 0.9510; 95% CI, 0.9329–0.9693; *p* < 0.001).

**Figure 3:**
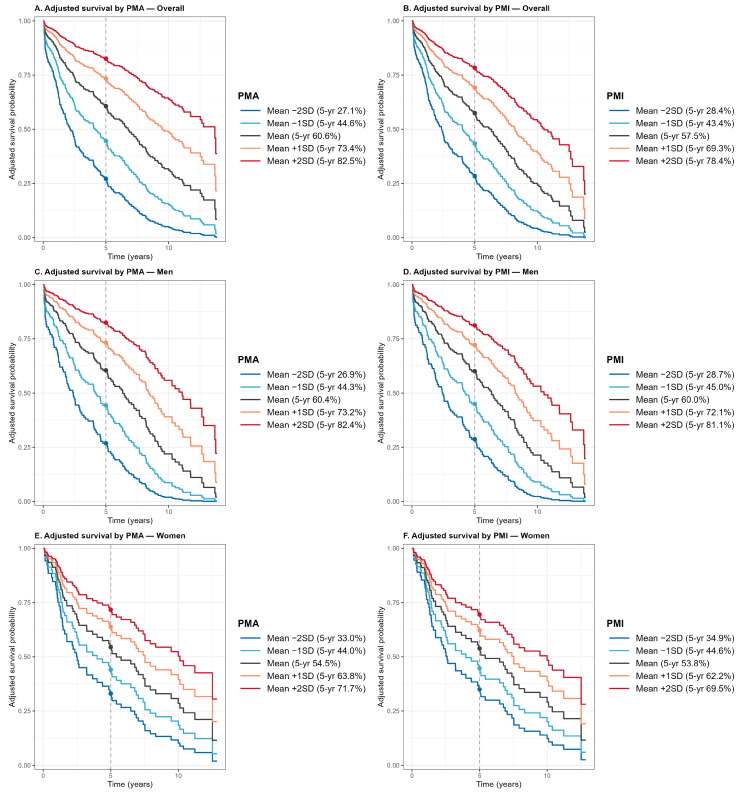
Adjusted survival curves by PMA and PMI across the overall cohort and sex-stratified subgroups. (**A**,**C**,**E**) PMA as the exposure; (**B**,**D**,**F**) PMI as the exposure. (**A**,**B**) Overall cohort; (**C**,**D**) men; (**E**,**F**) women. Curves represent predicted survival from multivariable Cox proportional hazards models at exposure values of mean −2 SD, −1 SD, mean, +1 SD, and +2 SD, with the mean and SD computed within the relevant analysis sample. All other covariates—age, hypertension, diabetes, asthma, coronary artery disease, cardiac disease, cancer, and inhaler use (plus sex in panels **A**,**B**)—were held constant at their sample mean values; for binary covariates, the mean corresponds to the observed prevalence. Vertical dashed lines indicate the 5-year time point; colored markers and legend values denote the predicted 5-year survival probability for each exposure level. Abbreviations: PMA, pectoralis muscle area; PMI, pectoralis muscle index; SD, standard deviation.

In the multivariable Cox proportional hazards model including PMI, higher PMI was likewise significantly associated with a lower risk of all-cause mortality (HR per 100-unit increase, 0.8786; 95% CI, 0.8343–0.9253; *p* < 0.001) (Table 2). The pectoralis muscle segmentation method is illustrated in Figure 2. The corresponding adjusted survival curves are presented in Figure 3A (PMA) and Figure 3B (PMI). When all covariates were held at their mean values (with binary covariates set to their observed prevalence), the estimated 5-year survival probability of the overall cohort was 60.6% for the PMA model and 57.5% for the PMI model. A one standard deviation decrease in PMA from the mean was associated with a substantial reduction in the predicted 5-year survival probability (to 44.6%), whereas a one standard deviation increase was associated with a corresponding improvement (to 73.4%) (Figure 3A). A comparable gradient was observed across PMI levels (Figure 3B; 5-year survival, 43.4% at −1 SD and 69.3% at +1 SD).

### 3.3. Sex-Stratified Analysis

In sex-stratified analyses, both PMA and PMI were significantly associated with lower mortality in men (PMA: HR per 100 mm^2^ increase, 0.9502; 95% CI, 0.9306–0.9702; *p* < 0.001; PMI: HR per 100-unit increase, 0.8731; 95% CI, 0.8239–0.9253; *p* < 0.001). In women, neither PMA nor PMI was significantly associated with mortality (PMA: HR per 100 mm^2^ increase, 0.9479; 95% CI, 0.8868–1.0131; *p* = 0.115; PMI: HR per 100-unit increase, 0.8991; 95% CI, 0.7782–1.0386; *p* = 0.148). Sex-stratified adjusted survival curves are shown in Figure 3C (PMA in men), Figure 3D (PMI in men), Figure 3E (PMA in women), and Figure 3F (PMI in women). In men, the curves demonstrated a clear separation across exposure levels: with all other covariates held at the male-subgroup means, the estimated 5-year survival probabilities for PMA were 60.4% at the mean, 44.3% at −1 SD, and 73.2% at +1 SD (Figure 3C), and the corresponding values for PMI were 60.0%, 45.0%, and 72.1%, respectively (Figure 3D). In women, the curves overlapped more substantially and the gradient across PMA and PMI levels was visually attenuated, consistent with the non-significant hazard ratios obtained in the regression analyses. The estimated 5-year survival probabilities at the mean, −1 SD, and +1 SD were 54.5%, 44.0%, and 63.8% for PMA (Figure 3E), and 53.8%, 44.6%, and 62.2% for PMI (Figure 3F).

### 3.4. Sensitivity Analysis

To evaluate the robustness of our findings under fewer modeling assumptions, we performed a sensitivity analysis using a random survival forest, which does not rely on the proportional hazards or linearity assumptions inherent to Cox regression. PMA and PMI were entered as the exposure of interest in separate models, with the same confounders as in the primary Cox analyses.

Out-of-bag C-indices from RSF were comparable to those from the Cox models in the overall cohort and in men, indicating that the prognostic ranking obtained from the Cox models was reproduced under a non-parametric framework; in women, the RSF C-index was lower than that of Cox, likely reflecting model instability in the smaller subgroup rather than a substantive difference in the underlying association (Table 3). VIMP analyses identified PMA and PMI as among the most influential predictors of mortality, with VIMP values comparable to or higher than that of age in the overall cohort and in men (Table 3; full VIMP results for all covariates are provided in Supplementary Table S1).

## 4. Discussion

In this study, CT-derived pectoralis muscle measurements were significantly associated with all-cause mortality in patients with COPD. Both absolute muscle area and height-adjusted indices demonstrated consistent prognostic value, suggesting that pectoralis muscle quantification on routine chest CT may serve as a useful imaging biomarker for risk stratification.

The association between lower pectoralis muscle measurements and higher mortality is biologically plausible and aligns with the concept of COPD as a systemic disease with clinically important extrapulmonary manifestations, including skeletal muscle depletion [28]. COPD is increasingly recognized as a systemic disease in which muscle loss reflects the combined effects of chronic inflammation, oxidative stress, physical inactivity, nutritional imbalance, and metabolic dysregulation [29,30]. These processes may reduce physiologic reserve, contribute to frailty, and increase susceptibility to adverse clinical outcomes [31]. Previous studies have shown that reduced skeletal muscle mass is associated with poorer prognosis, functional decline, and increased mortality in COPD and other chronic diseases [32,33]. In this context, CT-based pectoralis muscle assessment may serve as an objective imaging biomarker of systemic vulnerability that is not fully captured by conventional clinical variables alone [34]. In line with these findings, CT-derived pectoralis muscle measurements were associated with disease severity and adverse clinical outcomes in COPD. However, unlike previous studies that primarily focused on cross-sectional associations or functional parameters, our study extends these findings by demonstrating a clear association with long-term all-cause mortality.

A key finding of this study is the concordance between PMA and PMI. Although PMI accounts for body size by incorporating height, both metrics showed similar directions of association with mortality. This consistency suggests that the observed prognostic signal is robust across different approaches to muscle quantification and is primarily related to reduced muscle quantity itself rather than to the choice of normalization method. Prior studies have also suggested that both absolute and normalized muscle indices can provide meaningful prognostic information in chronic respiratory and systemic diseases [35,36]. However, our study further highlights the concordance between PMA and PMI in relation to mortality, supporting the robustness of pectoralis muscle measurements regardless of the normalization approach.

Another important observation was the sex-specific pattern identified in the stratified analyses. In men, both PMA and PMI were significantly associated with mortality, whereas neither metric reached statistical significance in women. Several factors may account for this difference. Sex-related variation in body composition, muscle reserve, fat distribution, and the clinical expression of COPD may influence the relationship between thoracic muscle measurements and outcomes [20,37]. Hormonal and metabolic factors may also modify the consequences of muscle depletion differently between men and women [21,38]. However, this finding should be interpreted with caution. The relatively small number of women in our cohort may have limited statistical power, potentially contributing to the lack of statistical significance despite similar trends. Therefore, these results should be considered hypothesis-generating rather than definitive evidence of sex-based effect modification. Previous studies have reported sex-related differences in muscle composition and in the clinical impact of sarcopenia in COPD, although these findings have not been entirely consistent across cohorts and definitions [39]. Consistent with the primary Cox analyses, the sensitivity analysis using a random survival forest demonstrated robust prognostic contributions of PMA and PMI in the overall cohort and in men, while results in women were less stable, in line with the smaller subgroup size and the attenuated gradient observed in the adjusted survival curves. Notably, in the variable importance analysis, PMA and PMI consistently outranked the available non-imaging clinical variables (comorbidities and inhaler use), highlighting the prognostic weight of chest CT-derived body-composition information relative to routinely collected clinical data. Importantly, these findings also indicate that the prognostic information carried by PMA and PMI is robustly captured by a non-parametric machine-learning model and is not an artifact of the parametric assumptions used in Cox regression. Beyond confirming the validity of our primary findings, this further suggests that PMA and PMI carry sufficient predictive signal to serve as candidate input features in future machine-learning-based prognostic models for COPD. Such an integration is particularly plausible because PMA and PMI are quantitative parameters obtained from the same chest CT examination already used for COPD assessment; for example, they could complement existing deep learning-based imaging biomarkers of lung parenchymal abnormality, such as the anomaly detection framework recently proposed for chest CT in COPD [40,41] by adding extrapulmonary, body-composition information from the same scan without additional acquisition.

Our findings may have broader clinical implications. Muscle depletion identified on routine chest CT may help recognize patients with COPD who are at increased risk and who may have a greater burden of extrapulmonary disease [42]. Sarcopenia and physical deconditioning are clinically important and have been targets of interventions such as pulmonary rehabilitation, exercise training, and nutritional support [43,44]. These interventions have been investigated for their potential to improve muscle status, physical performance, and broader clinical outcomes [45,46]. In this context, CT-based muscle assessment may contribute to a more integrated risk assessment framework in COPD by providing additional information on extrapulmonary disease burden.

Several limitations should be acknowledged. First, the retrospective design introduces the possibility of selection bias and unmeasured confounding. Second, this was a single-center study, which may limit generalizability. Third, although muscle measurements were obtained using a standardized semi-automated method with radiologist review and independent verification, reproducibility may vary across software platforms, segmentation algorithms, and CT acquisition parameters. Additionally, subtle or subclinical abnormalities of the pectoralis muscles, such as mild degeneration, unrecognized prior injury, or fatty infiltration related to degenerative musculoskeletal conditions, could not be completely excluded, which may have influenced the muscle measurements. Furthermore, interobserver agreement was not formally assessed, which may limit the evaluation of measurement reproducibility. Fourth, chest CT is not routinely required for the diagnosis of COPD, and the timing of CT acquisition was not fully standardized in relation to disease onset, which may have introduced heterogeneity in baseline imaging assessment. However, because COPD is a chronic and slowly progressive disease, the structural lung changes and muscle measurements assessed on CT are unlikely to be substantially affected by this temporal discrepancy. Thus, the impact of this limitation on the overall findings is expected to be minimal. Finally, the relatively small number of women may have reduced the power of the sex-stratified analyses.

## 5. Conclusions

CT-derived pectoralis muscle measurements, including both PMA and PMI, were significantly associated with all-cause mortality in patients with COPD. These findings support the potential role of opportunistic thoracic muscle quantification on routine chest CT as a clinically relevant imaging biomarker for prognostic stratification in COPD. Further studies are warranted to validate these findings in larger and more diverse populations, to determine their incremental value beyond established prognostic markers, and to clarify how CT-based muscle assessment can be integrated into clinical risk stratification.

## Figures and Tables

**Table 1:** Baseline characteristics of COPD patients.

Variable	Women (*n* = 81) ^1^	Men (*n* = 164) ^1^	Total (*n* = 245) ^1^	*p*-Value ^2^
Age (years)	73 ± 12	71 ± 10	72 ± 10	0.35
Follow-up duration (years)	5.1 ± 3.9	5.4 ± 4.0	5.3 ± 3.9	0.54
All-cause mortality	55 (67.9%)	123 (75.0%)	178 (72.7%)	0.31
PMA (mm^2^)	2179 ± 565	3163 ± 939	2838 ± 953	<0.001
PMI (index)	974 ± 249	1164 ± 329	1101 ± 318	<0.001
HTN	36 (44.4%)	58 (35.4%)	94 (38.4%)	0.22
DM	23 (28.4%)	34 (20.7%)	57 (23.3%)	0.24
Asthma	34 (42.0%)	34 (20.7%)	68 (27.8%)	<0.001
Coronary artery disease	15 (18.5%)	20 (12.2%)	35 (14.3%)	0.26
Cardiac disease	22 (27.2%)	27 (16.5%)	49 (20.0%)	0.07
Cancer	10 (12.3%)	18 (11.0%)	28 (11.4%)	0.92
Inhaler use	71 (87.7%)	141 (86.0%)	212 (86.5%)	0.87

^1^ Mean ± SD; *n* (%). ^2^ Welch Two Sample *t*-test; Pearson’s chi-squared test. PMA, pectoralis muscle area; PMI, pectoralis muscle index; HTN, hypertension; DM, diabetes mellitus; Cancer, any history of solid or hematologic malignancy; Inhaler use, any prescribed inhaled medication. Follow-up duration was defined as the interval from baseline chest CT to death or the last follow-up.

**Table 2:** Multivariable Cox regression analysis for all-cause mortality in COPD.

Subgroup	Measurement	HR (95% CI)	*p*-Value
Whole cohort (*n* = 245)	PMA (per 100 mm^2^ increase)	0.9510 (0.9329–0.9693)	<0.001
Whole cohort (*n* = 245)	PMI (per 100-unit increase)	0.8786 (0.8343–0.9253)	<0.001
Men (*n* = 164)	PMA (per 100 mm^2^ increase)	0.9502 (0.9306–0.9702)	<0.001
Men (*n* = 164)	PMI (per 100-unit increase)	0.8731 (0.8239–0.9253)	<0.001
Women (*n* = 81)	PMA (per 100 mm^2^ increase)	0.9479 (0.8868–1.0131)	0.12
Women (*n* = 81)	PMI (per 100-unit increase)	0.8991 (0.7782–1.0386)	0.15

HR, hazard ratio; CI, confidence interval; PMA, pectoralis muscle area; PMI, pectoralis muscle index. All models were adjusted for age, sex, hypertension, diabetes, asthma, coronary artery disease, cardiac disease, cancer, and inhaler use. Hazard ratios are expressed per 100-unit increase for PMA (mm^2^) and PMI.

**Table 3:** Comparison of multivariable Cox proportional hazards model and random survival forest for mortality prediction by PMA and PMI.

Subgroup	Exposure	*n*	Events	Cox C-Index	RSF C-Index	VIMP
Overall	PMA	245	178	0.668	0.636	0.153
Overall	PMI	245	178	0.664	0.640	0.177
Men	PMA	164	123	0.676	0.633	0.180
Men	PMI	164	123	0.669	0.635	0.173
Women	PMA	81	55	0.665	0.569	0.131
Women	PMI	81	55	0.662	0.559	0.115

Cox models and RSF were fitted with the same covariate set (age, sex [overall model only], hypertension, diabetes, asthma, coronary artery disease, cardiac disease, cancer, and inhaler use). RSF was run with 1000 trees. The C-index for RSF is the out-of-bag concordance index. VIMP denotes the permutation-based variable importance for the main exposure within each model (higher values indicate greater contribution to mortality prediction). Abbreviations: PMA, pectoralis muscle area; PMI, pectoralis muscle index; RSF, random survival forest; C-index, concordance index; VIMP, variable importance.

## Data Availability

The data are not publicly available due to privacy restrictions but are available from the corresponding author upon reasonable request.

## Supplementary Materials

The following supporting information can be downloaded at: https://www.mdpi.com/article/10.3390/diagnostics16111645/s1, Table S1: Permutation-based variable importance (VIMP) from random survival forest models for all covariates by subgroup and primary exposure.

## Abbreviations

The following abbreviations are used in this manuscript:

COPD	Chronic Obstructive Pulmonary Disease
CT	Computed Tomography
PMA	Pectoralis Muscle Area
PMI	Pectoralis Muscle Index
HR	Hazard Ratio
CI	Confidence Interval
GOLD	Global Initiative for Chronic Obstructive Lung Disease
HTN	Hypertension
DM	Diabetes Mellitus
SD	Standard Deviation
RSF	Random Survival Forest
VIMP	Variable Importance
