# Supplementary material for: CT-Derived Pectoralis Muscle Measurements and All-Cause Mortality in COPD
Source: Diagnostics (Basel). 2026 May 27;16(11):1645. doi: 10.3390/diagnostics16111645 (PMC13257103; doi:10.3390/diagnostics16111645)
Supplement: Supplementary file 1 [file diagnostics-16-01645-s001.zip › diagnostics-4318631-supplementary.pdf]

Table S1. Permutation-based variable importance (VIMP) from random survival forest (RSF) for all covariates, by subgroup and primary exposure.

Variable	Overall (N = 245)		Men (N = 164)		Women (N = 81)	
	PMA	PMI	PMA	PMI	PMA	PMI
**PMA**	**0.153**	—	**0.180**	—	**0.131**	—
**PMI**	—	**0.177**	—	**0.173**	—	**0.115**
age	0.113	0.113	0.089	0.098	-0.021	-0.008
sex	0.011	0.003	—	—	—	—
hypertension	0.004	0.003	0.002	0.002	-0.004	-0.003
diabetes	0.001	0.000	0.001	-0.001	0.057	0.059
asthma	-0.001	-0.001	-0.002	-0.003	-0.001	-0.002
coronary artery disease	-0.001	0.000	0.003	0.004	-0.004	-0.004
cardiac disease	0.021	0.019	0.008	0.009	0.022	0.025
cancer	0.012	0.009	0.017	0.016	-0.003	-0.005
inhaler use	-0.001	-0.002	-0.004	-0.004	0.002	0.000

Values represent permutation-based variable importance (VIMP) estimated by random survival forest (1,000 trees; fixed random seed = 2026; randomForestSRC package). Higher values indicate greater contribution to mortality prediction, whereas values near zero (including small negative values) indicate negligible contribution. The sign of VIMP does not indicate the direction of association. Dashes (—) indicate variables not included in the corresponding model; sex was used to define strata in sex-specific models. PMA and PMI were entered as the primary exposures in separate models to mirror the Cox analyses. Main exposures (PMA, PMI) are shown in bold. Abbreviations: PMA, pectoralis muscle area; PMI, pectoralis muscle index; RSF, random survival forest; VIMP, variable importance.
